# Trunk and Shoulder Kinematic and Kinetic and Electromyographic Adaptations to Slope Increase during Motorized Treadmill Propulsion among Manual Wheelchair Users with a Spinal Cord Injury

**DOI:** 10.1155/2015/636319

**Published:** 2015-02-22

**Authors:** Dany Gagnon, Annie-Claude Babineau, Audrey Champagne, Guillaume Desroches, Rachid Aissaoui

**Affiliations:** ^1^School of Rehabilitation, Université de Montréal, Montreal, QC, Canada H3C 3J7; ^2^Pathokinesiology Laboratory, Centre for Interdisciplinary Research in Rehabilitation of Greater Montreal, Institut de Réadaptation Gingras-Lindsay-de-Montréal, 6300 Darlington, Montreal, QC, Canada H3S 2J4; ^3^Department of Automated Production Engineering, École de Technologie Supérieure, Montreal, QC, Canada H3C 1K3

## Abstract

The main objective was to quantify the effects of five different slopes on trunk and shoulder kinematics as well as shoulder kinetic and muscular demands during manual wheelchair (MWC) propulsion on a motorized treadmill. Eighteen participants with spinal cord injury propelled their MWC at a self-selected constant speed on a motorized treadmill set at different slopes (0°, 2.7°, 3.6°, 4.8°, and 7.1°). Trunk and upper limb movements were recorded with a motion analysis system. Net shoulder joint moments were computed with the forces applied to the handrims measured with an instrumented wheel. To quantify muscular demand, the electromyographic activity (EMG) of the pectoralis major (clavicular and sternal portions) and deltoid (anterior and posterior fibers) was recorded during the experimental tasks and normalized against maximum EMG values obtained during static contractions. Overall, forward trunk flexion and shoulder flexion increased as the slope became steeper, whereas shoulder flexion, adduction, and internal rotation moments along with the muscular demand also increased as the slope became steeper. The results confirm that forward trunk flexion and shoulder flexion movement amplitudes, along with shoulder mechanical and muscular demands, generally increase when the slope of the treadmill increases despite some similarities between the 2.7° to 3.6° and 3.6° to 4.8° slope increments.

## 1. Introduction

There has been a growing interest in motorized treadmill manual wheelchair (MWC) propulsion in recent years in rehabilitation research environments, and to a lesser extent in clinical practice, since it seems to closely duplicate overground MWC requirements [1]. Motorized treadmill MWC propulsion also allows for propulsion, in a restricted space, during short (e.g., high intensity interval training) and prolonged (e.g., cardiorespiratory fitness training) periods of time at different speed or slope parameters. Moreover, unlike propulsion on a roller ergometer or a dynamometer, MWC propulsion on a motorized treadmill allows for greater freedom of MWC movements and some inertial effect exposure linked to the acceleration/deceleration of the wheelchair and head-trunk-upper limb segments. Hence, motorized treadmill MWC propulsion is a promising therapeutic alternative founded on the principle of repetitive task-specific training and anticipated sensorimotor adaptations. The quality and quantity of evidence currently available on motorized MWC propulsion do not inform rehabilitation professionals about how to vary parameters that can be easily modulated (e.g., the speed or slope of the treadmill) and their effects during motorized treadmill MWC propulsion. Stronger evidence is needed to optimize assessment and training protocols.

Propelling a MWC up slopes on a motorized treadmill has been found to increase upper limb demand in a few recent studies. Richter et al. [2] found among MWC users with a spinal cord injury (SCI), propelling at a self-selected velocity on a motorized treadmill, that the speed was about 1.5 and 2.7 times slower when pushing up 3° and 6° slopes, respectively, in comparison with the level surface (0° slope). Additionally, they also reported that the peak total force at the handrim was about 1.7 and 2.2 times higher when pushing up 3° and 6° slopes, respectively, in comparison with the level surface (0° slope) despite the reduced treadmill speed. Yang et al. [3] found among MWC users with a SCI, propelling at an imposed steady speed of 0.9 m/s on a motorized treadmill, that peak shoulder flexion increased by 9° while the total and tangential forces applied at the handrim were about 2.09 and 2.38 times higher when pushing up a 3° slope (approximately a 1 : 20 ratio) in comparison with the level surface, whereas the mechanical efficiency was only found to be 1.1 times higher. More recently, Gagnon et al. [4] found among MWC users with a SCI, each propelling at a self-selected steady speed on a motorized treadmill up slopes set at 0°, 2.7°, 3.6°, 4.8°, and 7.1°, that the total and tangential forces applied at the handrim were at least 2 times greater as the slope became progressively steeper. The greatest change observed was between 0° and 2.7°, while similarities were observed between 2.7° and 3.6°. Such differences also support the need to gain a better understanding of the effects of steeper slopes on trunk and shoulder kinematics, shoulder kinetics, and shoulder muscular demand during uphill propulsion on a treadmill set at a steady speed among a group of experienced MWC users.

In addition, few studies have investigated the effects of varying slopes during overground MWC propulsion over the past decade. Among those, Chow et al. [5] investigated the effects of various slopes (i.e., 0°, 2°, 4°, 6°, 8°, 10°, and 12°) on trunk kinematics, handrim kinetics, and upper limb and upper trunk muscular demand among MWC users with a spinal cord injury. Overall, forward trunk flexion and muscular demand (i.e., triceps brachii, anterior deltoid, and pectoralis major) were found to progressively increase as the slope became steeper. van Drongelen et al. [6] compared shoulder net joint moments between overground level and uphill (i.e., 3° slope) propulsion among MWC users with paraplegia and tetraplegia. The resultant net shoulder moments were about 2 times higher during uphill compared to overground level MWC propulsion. Arabi et al. [7] compared relative mechanical demand during uphill propulsion across three slopes (i.e., 2.7°, 4.8°, and 5.7°) among a group of able-bodied individuals who used a MWC. They confirmed that the relative mechanical demand significantly increased as the slope became steeper and reached 16.1%, 25.7%, and 31.1% of their maximum isometric voluntary force generating capability for the three slopes tested, respectively. Last, Kulig et al. [8] compared shoulder net joint moments between level overground and uphill (slope = 4.7°) MWC propulsion in individuals with paraplegia. They showed that the peak net shoulder flexion, adduction and internal rotation moments were 2.2, 2.2, and 2.7 times greater, respectively, when ascending a slope compared to level overground wheelchair propulsion.

The aim of this study was to quantify the trunk and nondominant shoulder kinematic changes along with the nondominant shoulder joint moments and electromyographic changes during MWC propulsion on a motorized treadmill set at a self-selected natural speed on five different slopes (i.e., 0°, 2.7°, 3.6°, 4.8° and 7.1°). It was expected that the nondominant shoulder and trunk range of motion and nondominant shoulder joint moments and muscle electromyographic activity would gradually and significantly increase with each slope increment while the speed remained constant.

## 2. Material and Methods

### 2.1. Description of Participants

A convenience sample of 17 men and 1 woman who sustained a SCI (American Spinal Injury Association Impairment Scale [9] (AIS) = A, B, C or D) volunteered to participate in this study (Table 1). Participants were included in the study if they had sustained a SCI at least three months before the study, had been discharged from initial intensive inpatient rehabilitation, were living in the community and used their MWC for at least 4 hours per day. Participants also had to master basic and advanced wheelchair skills, including the capability to propel up a 9-metre long access ramp meeting building code standards in the province of Quebec in Canada (i.e., maximum slope of 1 : 12 for slopes of a maximum length of 9 metres) [10]. Participants were excluded if they presented associated neurological conditions, musculoskeletal impairments/pain, cardiorespiratory/vascular conditions, or any other impairments or disabilities that might have interfered with the performance or safety of the experimental tasks. The self-reported Wheelchair User's Shoulder Pain Index (WUSPI) questionnaire [11, 12] was completed (group's mean score = 0.89 ± 1.05/10) and reviewed by a physical therapist who asked specific questions whenever pain was rated as interfering with the performance of wheelchair mobility to further verify that pain will not limit their ability to specifically complete the experimental tasks. During a telephone interview with the potential participants, the rehabilitation research coordinator reviewed the inclusion and exclusion criteria to determine eligibility before scheduling the clinical and laboratory assessments. All participants gave their written consent to participate in the study after being informed of the objectives and nature of their participation in the study. The Research Ethics Committee of the Centre for Interdisciplinary Research in Rehabilitation of Greater Montreal (CRIR #715-0312) approved the present study.

### 2.2. Clinical Evaluation

Each participant underwent a clinical assessment, completed by a physical therapist, in order to collect their personal characteristics, measure their anthropometric parameters (height, weight, length, and circumference of body segments), characterize the severity of the sensory and motor impairments (ASIA Impairment Scale [13]), confirm the absence of debilitating U/L musculoskeletal impairment (i.e., WUSPI [11, 12], U/L joint ranges of motion, U/L static manual muscle testing), and confirm U/L nondominance [9].

### 2.3. Motorized Treadmill Wheelchair Propulsion

**Figure 1:**
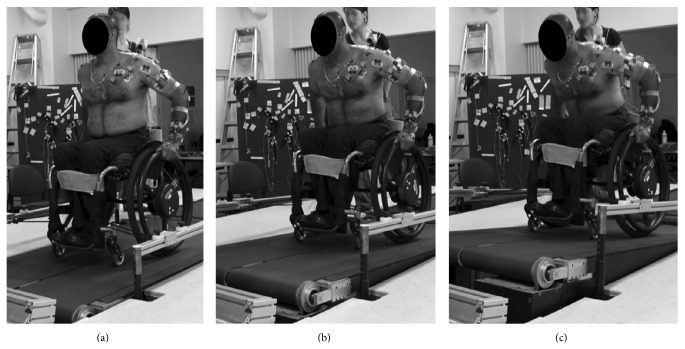
Illustration of three slopes tested during motorized treadmill MWC propulsion: (a) 0° slope, (b) 3.6° slope, and (c) 7.1° slope.

At the start of the laboratory assessment, each participant was given a five-minute familiarization period of motorized treadmill propulsion at various slopes that differed from those investigated in the present study during which rest periods were allowed to avoid fatigue. The motorized dual belt instrumented treadmill (Bertec Corporation, Columbus, Ohio, United States) (width = 0.84 m; length = 1.84 m) was adapted for safe MWC propulsion. The MWC was anchored with elastic bands to a bilateral frictionless gliding safety system preventing excessive antero-posterior and rotational movements of the MWC (Figure 1). The imposed speed of the treadmill was adjusted for each participant to mimic the self-selected natural propulsion speed measured during a timed performance-based 20 meter MWC propulsion test. This last test was performed three times with a two-minute rest taken between trials to compute the self-selected natural propulsion speed. Thereafter, each participant first propelled their own MWC on the motorized treadmill with a level ground (0°) and then randomly at four different slopes: 2.7°, 3.6°, 4.8°, and 7.1°, reflecting an increase from one unit of height to 20, 16, 12, and 8 units of length, respectively. For each angle tested, two trials lasting a maximum of one minute (i.e., 20 consecutive pushes) and separated by a two-minute rest period were recorded. During each trial, the last 10 complete consecutive propulsion cycles recorded were used to compute the measurements of interest (i.e., trunk and shoulder kinematics, shoulder kinetics, and shoulder muscular demand) and were essential to confirm the successful completion for each slope tested (i.e., two trials/slope). This study design was selected to minimize systematic errors related to the testing (e.g., learning) and temporary maturation effects (e.g., fatigue) associated with the experimental protocol and to conclude that the findings of the present study do not result from these potential threats to internal validity. Participants rated their perceived nondominant localized U/L effort using a 10 cm visual analog scale ranging from* “no effort”* (0 cm) to* “maximum effort”* (10 cm) during the rest periods.

### 2.4. Trunk and Shoulder Kinematics

To capture the 3D movements of the trunk, the nondominant U/L and the MWC, a total of 27 skin-fixed light-emitting diodes (LEDs) were placed on specific anatomical landmarks while four LEDs were fixed to the MWC frame [14]. The 3D coordinates of each LED within the laboratory coordinate system were collected at 30 Hz with a motion capture system incorporating four synchronised camera units (Optotrack 3020 and Optotrack Certus; Northern Digital Inc., Waterloo, Ontario, Canada, http://www.ndigital.com). Supplementary bony landmarks, wheelchair, and treadmill reference points were digitised to determine principal axes of segments and locate articular joint centres for the trunk and nondominant U/L, wheelchair position and treadmill slope. Before initiating the experimental tasks, three abduction-adduction and three flexion-extension active movements were recorded to locate the shoulder articular centre with respect to the scapula using a quadratic sphere fitting procedure [15, 16]. All marker trajectories were visually inspected and interpolated when coordinates were missing using a linear or a cubic spline method. The marker trajectories were then filtered using a 4th order zero-lag Butterworth filter with a cutoff frequency set at 6 Hz. The recommendations formulated by the International Society of Biomechanics [17] were used to determine segmental coordinate systems (head, trunk, arms, forearms, and hand). Relative motion between the humerus and clavicle, used as a surrogate rigid segment for the scapula that articulates with the humerus [17], was computed using a ZX′Y′′ cardanic rotation sequence to avoid gimbal lock and to interpret reconstructed shoulder movements according to three anatomical movements commonly described in clinical practice (i.e., flexion/extension, abduction/adduction, internal and external rotations) [18]. The relative trunk forward inclination angle (i.e., forward trunk flexion) was computed as the motion of the vertical axis of the trunk, defined by a unit vector created with the midpoint between the eighth thoracic vertebra and the xiphoid process to the midpoint between the seventh cervical vertebra and the sternal notch, with respect to the vertical axis of the laboratory coordinate system. For the trunk forward flexion/extension, and the shoulder flexion/extension, adduction/adduction, and internal/external rotation, the minimal and maximal movements along with their total excursion were the main outcome measures.

### 2.5. Handrim Kinetics

Each participant's MWC was equipped bilaterally with 24′′ or 26′′ instrumented wheels (SmartWheel (SmartWheel, Out-Front (formerly Three Rivers Holdings, LLC), Mesa, Arizona, United States, http://www.out-front.com/)) to measure the three dimensional components of the total force applied at the handrim during MWC propulsion at a sampling frequency of 240 Hz [19]. While these instrumented wheels did not alter axle position or other rear wheel spatial characteristics (e.g., orientation and diameter of the handrim), they slightly increased wheelchair width and weight (4.8 kg/instrumented wheel) and may have affected rolling resistance (i.e., urethane tire). Three dimensional handrim kinetic data were filtered with a 4th order Butterworth filter and a cutoff frequency of 20 Hz and then downsampled to 30 Hz to fit the kinematic data using a custom MATLAB (MATLAB, MathWorks, Natick, Massachusetts, United States, http://www.mathworks.com/) routine.

### 2.6. Shoulder Kinetics

Shoulder net joint moments were computed using an inverse dynamic method [20]. The data entered into a custom-made MATLAB algorithm included the anthropometric characteristics as well as the U/L kinematics and the pushrim kinetics with respect to the lab coordinate system. Shoulder net joint moments were then expressed in the same coordinate system used to express shoulder joint kinematics and normalized against the body mass of each participant. In fact, moderate to high associations were found between body mass and mean and peak shoulder net joint moments when propelling with no slope (*r* = 0.554 and 0.577) and with slopes of varying degrees (*r* = 0.713 to 0.809). The peak and mean shoulder net joint moments in the sagittal, frontal, and transverse plane were the main outcome measures.

### 2.7. Shoulder Muscular Demand

The electromyographic activity of the anterior and posterior portions of the deltoid along with the clavicular and sternal heads of the pectoralis major was recorded at the nondominant upper extremity at a sampling frequency of 1200 Hz using a portable telemetric system (NORAXON USA Inc.; Scottsdale, Arizona; Telemyo 900). Skin preparation and the placement of the surface electrodes (BlueSensor M, AMBU, Ballerup, Danmark) (Ag/AgCl sensor –13.2 mm² active surface area) were made in accordance with SENIAM recommendations (refer to http://www.seniam.org/). Following baseline noise removal, all EMG signals recorded were visually inspected before being filtered with a 4th order zero-lag Butterworth bandpass filter with low and high cut-off frequencies set at 30 and 500 Hz, respectively. Thereafter, EMG patterns were full-wave rectified and filtered with a 6 Hz low-pass filter to generate EMG linear envelopes for each muscle studied. The muscular utilization ratio (MUR (%)) was calculated for each muscle studied by normalizing the amplitude of the EMG signals recorded during the experimental tasks, against the peak EMG signal recorded over a 0.5 second period during one of the two static maximum voluntary contractions (MVC). Muscle-specific manual resistance was applied by a trained physiotherapist to generate the MVCs while participants remained seated in their own wheelchair. Meanwhile, another research associate manually provided additional trunk and wheelchair stability to participants. For each muscle studied, the peak and mean MURs as well as an indicator of muscle work (IMW), were calculated using the integral of the MUR data, were the main outcome measures. All EMG signal processing was performed digitally using a custom-developed MATLAB algorithm.

### 2.8. Statistical Analyses

Descriptive statistics (mean ± SD) were calculated for the demographic and clinical characteristics of all participants as well as for the kinematic, kinetic, and muscular demand outcome measures. For these last outcome measures, 10 propulsion cycles were averaged per trial resulting in a total of 20 propulsion cycles for each slope tested. The kinematic, kinetic and muscular demand data recorded during the push phase of each cycle analyzed were also time-normalized over 100% (i.e., 100 data points) to generate a profile for each participant and a mean group profile. Shapiro-Wilk tests confirmed that the kinematic, kinetic and electromyographic outcome measures for all slopes tested were normally distributed and justified the use of parametric statistical tests. One-way repeated-measure analyses of variance (ANOVA) with one within-subjects factor (slopes of 0°, 2.7°, 3.6°, 4.8°, and 7.1°) using a general linear model was used to determine the effect of the slopes on the kinematic and kinetic and electromyographic outcome measures and an eta-squared value was used to confirm if the proportion of the total variability attributable to the slope factor (i.e., effect size) was small (>0.02), moderate (>0.13), or large (>0.26). Whenever an ANOVA revealed significant differences (main effect; *P* < 0.05) after the result of the Mauchly's test of sphericity of the covariance matrix was taken into consideration, Student's *t*-tests for paired samples were computed (*post hoc* tests) with a Bonferroni correction setting the significance level at *P* ≤ 0.0125 (*P* ≤ 0.05/4 pairwise comparisons) as a result of the four possible slope increments (i.e., 0° to 2.7°, 2.7° to 3.6°, 3.6° to 4.8° and 4.8° to 7.1°). All statistical analyses were performed with SPSS Statistics 17.0 software for Windows. Note that the kinematic and electromyographic data were only collected and computed at the nondominant U/L since quasi-symmetrical U/L movement strategies and efforts were assumed in order to safely propel on a linear trajectory on the motorized treadmill [21] and since the nondominant U/L strength generating capability is generally weaker than the one at the dominant U/L, possibly resulting in higher relative demand at the nondominant U/L during the performance of a symmetrical functional task.

## 3. Results

### 3.1. Completion Rate

At a mean natural and constant self-selected propulsion speed of 1.17 ± 0.18 m/s [min = 0.91 m/s; max = 1.65 m/s], all participants (completion rate = 100%) were able to propel themselves on the 0° slope and up the 2.7° slope (Table 2). The completion rate reached 88.9% (*N* = 16/18 participants), 77.8% (*N* = 14/18 participants), and 55.6% (*N* = 10/18 participants) for the 3.6°, 4.8°, and 7.1° slopes, respectively (Table 2).

### 3.2. Temporal Parameters

Table 3 summarizes the mean duration of the push and recovery phases and the total duration of a propulsion cycle in seconds for the different treadmill slopes. The average durations of the push phase were similar for all tested slopes (ANOVA; *P* = 0.267), whereas the average duration of the recovery phase declined as the slope became steeper (*post hoc* tests; *P* ≤ 0.043). The total duration significantly decreased as the slope became steeper (*post hoc* tests; *P* ≤ 0.001), except during the 2.7° to 3.6° slope increment that remained similar.

### 3.3. Trunk and Shoulder Kinematics

**Figure 2:**
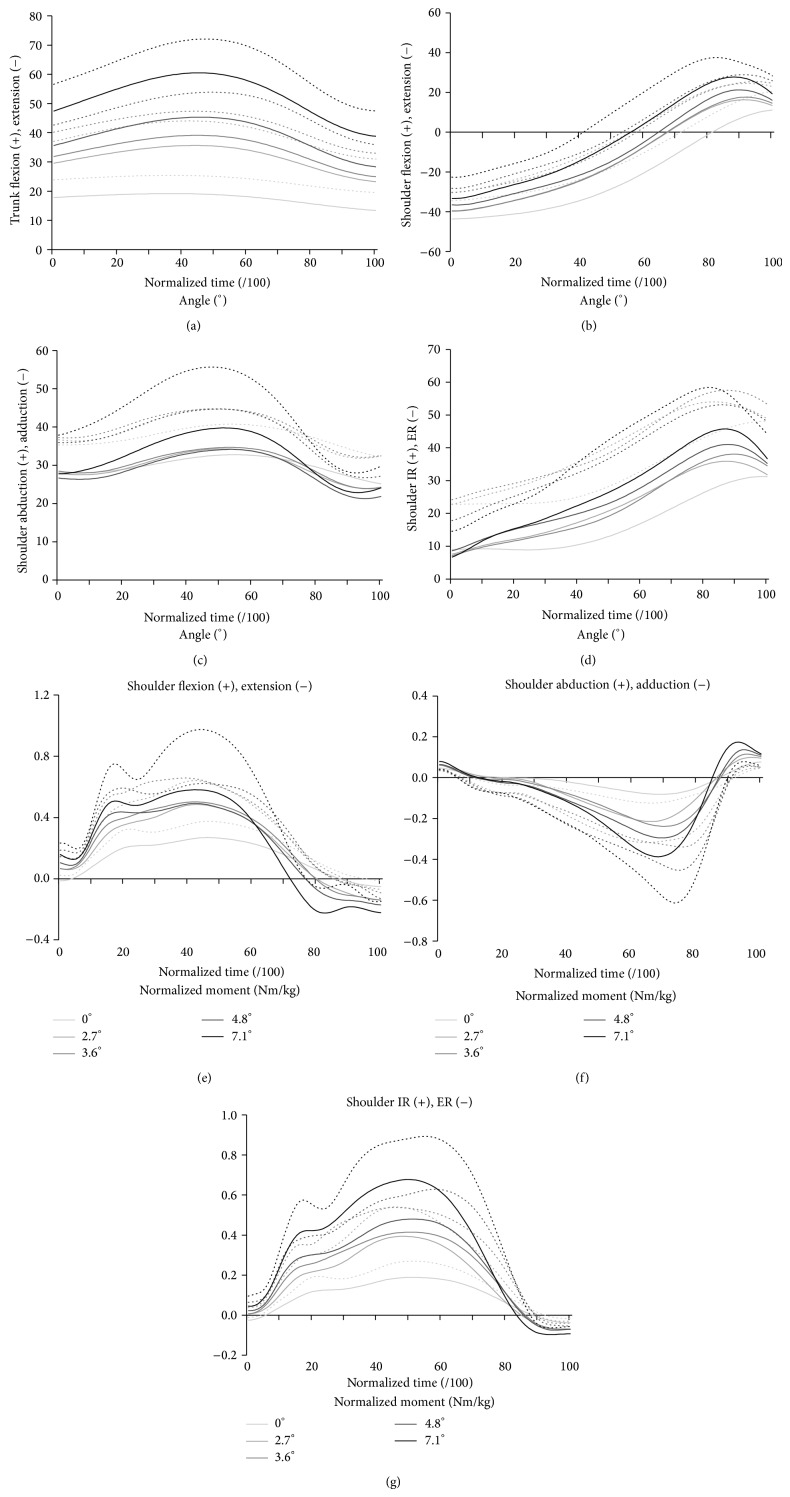
Group averaged time-normalized profile (solid line) and standard deviation (dotted line) of the shoulder and trunk kinematics (a, b, c, and d) and weight-normalised shoulder moments (e, f, and g) during the push phase for the five slopes tested at self-selected natural speed.

The trunk and shoulder movement patterns are illustrated in Figures 2(a), 2(b), 2(c), and 2(d), whereas the minimum, maximum, and excursion of the trunk and shoulder movement amplitudes are summarized in Table 4. The slopes of the treadmill significantly influenced most minimum, maximum, and excursion trunk and shoulder movement amplitudes. At the trunk, all minimum, maximum, and excursion movement amplitudes significantly increased as the slope became steeper, except for minimum and maximum values during the 2.7° to 3.6° slope increment that remained similar. The greatest maximum forward trunk flexion (60.9°), which was accompanied by the greatest forward trunk excursion (22.4°), was reached during the 7.1° slope. At the shoulder, the maximum shoulder flexion movement amplitude significantly increased as the slope became steeper, except for the 3.6° to 4.8° slope increment that remained similar, whereas the minimum shoulder flexion movement amplitude (i.e., shoulder extension) significantly decreased during that same period. As a result, the shoulder flexion excursion remained comparable despite slope increments. The minimum, maximum, and excursion shoulder abduction movement amplitudes remained comparable as the slope became steeper. The minimum, maximum and excursion shoulder internal rotation movement amplitudes also remained comparable as the slope became steeper, with the exception of the minimum and excursion values, which significantly increased during the 0° to 2.7° slope increment.

### 3.4. Shoulder Kinetics

The net shoulder flexion, abduction and internal rotation moment patterns are illustrated in Figures 2(e), 2(f), and 2(g) while their mean and maximum values are summarized in Table 5. The greatest maximum shoulder moments were found during flexion across all slopes except for the 7.1° slope when the internal rotation generated the greatest moment. All mean and maximum shoulder moments were significantly influenced by the slopes of the treadmill. The mean and maximum flexion moments significantly improved as the slope increased, except for the 3.6° to 4.8° and 4.8° to 7.1° slope increments. The mean adduction moments only significantly improved as the slope increased between 0° and 2.7°, whereas the peak mean value only significantly improved as the slope increased between the 0° to 2.7°, 3.6° to 4.8°, and 4.8° to 7.1° slope increments. The mean and maximum internal rotation moments significantly increased as the slope became steeper, except for the 3.6° to 4.8° slope increment.

### 3.5. Shoulder Muscular Demand

**Figure 3:**
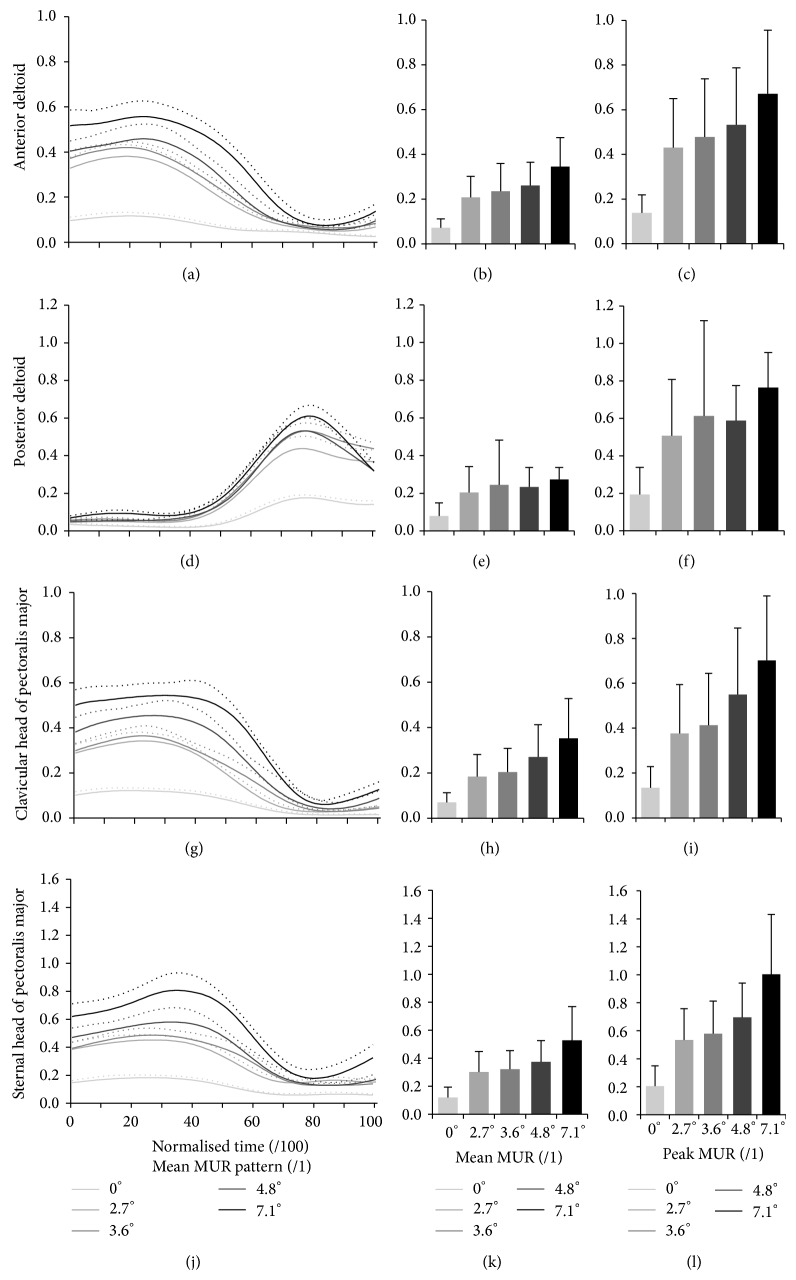
Group averaged time-normalized mean profile (solid line) and standard deviation (dotted line) (a, d, g, and j), as well as group-average (SD) mean (b, e, h, and k) and peak (c, f, i, and l) muscle utilization ratio (MUR) during the push phase for the five slopes tested at self-selected natural speed.

**Figure 4:**
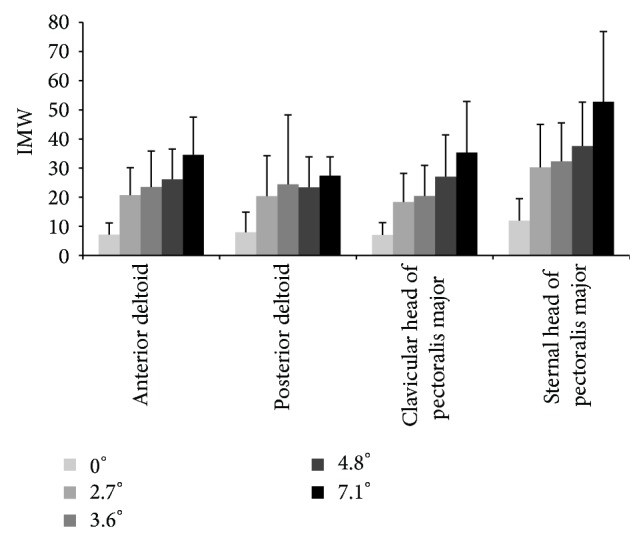
Group averaged (SD) mean indicator of muscle work (IMW) during the push phase for the five slopes tested at self-selected natural speed.

The MUR patterns of the muscles studied are illustrated in Figure 3, while their mean and maximum values are summarized in Table 6. The mean IMWs of the muscles studied are summarized in Table 6 and illustrated in Figure 4. For all muscles studied, their mean and maximum MURs, as well as their indicator of muscle work value, significantly increased as the slope became steeper, except for the posterior deltoid that remained comparable between the 2.7° to 3.6° slope increment.

## 4. Discussion

This study quantified trunk and nondominant shoulder kinematic changes along with nondominant shoulder joint moments and electromyographic changes during MWC propulsion on a motorized treadmill set at a self-selected natural speed on five different slopes (i.e., 0°, 2.7°, 3.6°, 4.8°, and 7.1°). Overall, the MWC users with a SCI increased forward trunk flexion and peak shoulder flexion while also increasing shoulder mechanical and muscular efforts to adapt to slopes that progressively increased during simulated uphill MWC propulsion on a motorized treadmill.

### 4.1. Trunk and Shoulder Movement-Related Adaptations

The movement-related adaptations occurring at the trunk and shoulder partially support the hypothesis that their outcome measures would gradually and significantly increase with each slope increment while the speed remained constant. At the trunk, the maximum forward trunk flexion and the total trunk excursion increased significantly as the slope became steeper, except for the 2.7° to 3.6° slope increment. Chow et al. [5] obtained somewhat comparable results in terms of trunk kinematics although no difference was revealed when comparing 4° and 8° slopes. This may be explained by the fact that participants propelled at self-selected speeds that decreased progressively in their protocol as the slope increased. Nonetheless, the increased maximum forward trunk flexion coupled with the increased forward trunk excursion may allow MWC users to move their centre of mass further and faster anteriorly and to maintain its projection in front of the rear wheel axle in order to prevent backward tilt and falls as the slope increases. This may also explain why maximum forward trunk flexion and forward trunk excursion became greater as the gravitational effects became harder to overcome with steeper slopes. The decreasing success rate with a steeper slope may be explained in part by the fact that some participants were classified as being overweight (body mass index > 25) or class I obese (body mass index = 30.0–34.9) with associated abdominal obesity that limited their ability to increase forward trunk flexion to accommodate for the steeper slopes. In fact, 55.6% of participants who were unable to propel up the 7.1° slope were overweight (*N* = 3) or obese (*N* = 2), whereas only 30% of participants who were able to propel up the 7.1° slope were overweight (*N* = 2) or obese (*N* = 1). Hence, abdominal circumference may deserve additional attention when investigating wheelchair propulsion technique or manual wheelchair skills such as uphill propulsion.

At the shoulder, the maximum shoulder flexion movement amplitude significantly increased as the slope became steeper, except for the 3.6° to 4.8° slope increment that remained similar, whereas the minimum shoulder flexion movement amplitude (i.e., shoulder extension) significantly decreased during that same period. As a result, the shoulder flexion excursion remained comparable despite slope increments. It is possible that increased maximum shoulder flexion was needed to accommodate for the increased forward trunk flexion and to apply most of the force tangentially on the handrim to preserve mechanical efficiency. The fact that only the duration of the recovery period drastically decreased as the slope became steeper can also explain, in part, the relatively stable shoulder flexion-extension excursion (i.e., similar push phase durations across slopes). This finding is consistent to some extent with the work of Yang et al. [3] who found comparable values of movement-related adaptation at the shoulder when comparing level overground and uphill (i.e., 3° slope) MWC propulsions. However, the shoulder flexion-extension excursion significantly increased on average by 9.37° and the shoulder extension remained similar in their study contrary to the results of the present study. These discrepancies may be attributed to the fact that participants used test wheelchairs that were not anchored to a safety system when propelling up a 3° slope at 0.9 m/s on a motorized treadmill, the level surface propulsion (0° slope) was performed overground, and the shoulder kinematic calculation differed from the work by Yang et al. [3]. Nevertheless, all these results confirmed kinematic adaptations at the shoulder as the slope progressed. Lastly, the increased forward trunk flexion coupled with an increased shoulder flexion that modifies the orientation of the force generated at the shoulder may increase posterior shoulder joint forces and explain the elevated muscular demand at the posterior deltoid occurring towards the end of the push phase [22].

### 4.2. Shoulder Joint Mechanical and Muscular Effort Adaptations

The shoulder joint moments adaptations partially support the hypothesis that their outcome measures would gradually and significantly increase with each slope increment while the speed remained constant. For two out of the three shoulder net joint moments investigated, most of the mean and peak values for the shoulder flexor and internal rotator moments progressively increased as the slope became steeper, aside from some outcome measures during the 2.7° to 3.6° or the 3.6° to 4.8° slope increment. The relative muscular demand adaptations and muscular work computed for all muscles investigated fully support this hypothesis since their outcome measures gradually and significantly increased with each slope increment, while the speed remained constant. Hence, once the results of these two approaches are combined, it is clear that the shoulder mechanical and muscular effort increases are key contributors to the adaptation process associated with the steeper slopes. Moreover, these results are in line with the perceived U/L efforts expressed by the participants and may explain, in part. The decreasing success rate with a steeper slope as shoulder strength generating capability most likely becomes a determinant for propelling on steeper slopes.

### 4.3. Implications for Clinical Practice

With the use of a slope or of a combination of slopes, when a MWC user propels himself/herself on a motorized treadmill, therapists may be able to offer task-specific high-intensity short duration interval training programs to increase U/L strength, particularly at the shoulders. Cautiousness is advised with this practice since the risk exposure (i.e., increased shoulder mechanical and muscular demands) will progressively and significantly increase as the slope becomes steeper and will vary according to the strength-generating capability of each MWC user. In addition, since the risk exposure could trigger the development or exacerbation of secondary impairments at the U/L, particularly at the shoulders, such a program should also be accompanied by proper warm-up and cool-down periods as well as by antagonist muscle strengthening to prevent muscle strength imbalance. Alternatively, therapists may also offer task-specific cardiorespiratory fitness training programs when a MWC user propels on a motorized treadmill with no slope or minimal slope (<2.7°) with minimal demands during a prolonged period of time (i.e., cardiorespiratory fitness training).

### 4.4. Limits of the Study

The present study included a relatively small sample size (*n* = 18) of experienced MWC users who have completed their rehabilitation process which may limit the strength of the evidence and potential to generalize the results with new MWCs, respectively. The fact that the participants used their personal wheelchairs during the study warrants consideration as optimal wheelchair positioning and configuration parameters most likely differs across participants and impacts the outcome measures of interest in the present study and the risk of the MWC tilting or falling backwards when propelling up a slope. The use of the instrumented wheels which slightly increase the width and weight of the wheelchair along with rolling resistance may have modified participants' propulsion technique (e.g., increased shoulder abduction) and fatigue level (e.g., increased U/L effort) in comparison to propelling with their own wheels. The self-selected natural treadmill speed determined for each participant, maintained across all slopes tested in an effort to isolate the effect of speed, also requires consideration since MWC users tend to reduce their speed when propelling uphill in daily life, particularly on steep slopes [2]. Finally, the kinematic and kinetic and electromyographic variables solely focused on the trunk and nondominant shoulder prevent a full understanding of U/L adaptations (i.e., elbow and wrist not studied) during motorized treadmill MWC propulsion across different slopes.

## 5. Conclusion

This study confirms that MWC users with a SCI increase forward trunk flexion and peak shoulder flexion while also increasing shoulder mechanical and muscular efforts to adapt to slopes that progressively increase during simulated uphill MWC propulsion on a motorized treadmill. Few similarities were found between the 2.7° to 3.6° and the 3.6° to 4.8° slope increments for shoulder flexion and adduction moments. Future studies incorporating interactions with various slopes and velocities could strengthen the results of the present study and provide additional evidence-based knowledge on wheelchair propulsion on a motorized treadmill.

## Figures and Tables

**Table 1:** Description of participants.

Participants	Gender	Age years	Height m	Mass kg	Time since injury years	ASIA^*^				WUSPI^*^		
						Neuro-logical level	AIS^*^	Sensory/224	Motor/100	Mean/10	Propulsion 10 min/10	Slope/10
1	M	44.3	1.84	80.3	10.6	T7	A	117	50	0.19	0.0	0.0
2	M	46.4	1.70	80.2	4.6	T10	B	140	50	0.68	0.1	0.5
3	M	32.2	1.92	95.9	5.3	T10	A	140	50	0.00	0.0	0.0
4	M	35.8	1.80	77.1	11.8	T6	D	194	81	1.25	1.9	2.2
5	M	33.2	1.95	72.3	7.8	T12	C	162	56	0.19	0.0	0.0
6	M	52.6	1.77	108.9	18.7	T9	A	132	50	1.14	2.4	3.4
7	M	59.9	1.88	99.8	5.0	T10	A	140	50	0.34	0.4	2.2
8	M	44.0	1.72	68.4	22.1	T4	B	183	35	0.07	0.9	0.0
9	M	41.2	1.78	72.7	6.1	C7	C	56	44	1.23	2.7	2.7
10	M	28.4	1.85	66.6	10.6	T12	A	154	50	0.63	3.8	1.9
11	M	39.0	1.76	101.8	2.8	T10	A	72	50	3.65	5.5	6.8
12	M	49.1	1.70	76.8	4.4	T7	A	88	52	0.97	1.5	2.4
13	M	55.7	1.80	103.1	4.9	T3	A	88	50	0.31	1.8	1.6
14	M	32.8	1.75	61.9	8.9	T4	A	95	50	0.10	0.4	0.4
15	F	28.1	1.65	47.5	4.8	T11	A	148	50	0.20	2.0	1.0
16	M	33.0	1.65	66.5	5.3	T6	A	53	50	0.10	0.0	0.3
17	M	52.7	1.73	78.2	8.9	T12	B	172	63	3.12	2.6	4.8
18	M	25.8	1.83	59.2	4.9	T7	A	112	50	1.88	4.7	2.6

Mean		**40.8**	**1.78**	**78.7**	**8.2**			**124.8**	**51.7**	**0.89**	**1.7**	**1.8**
SD		**10.3**	**0.09**	**17.0**	**5.1**			**42.3**	**9.0**	**1.05**	**1.7**	**1.9**

^*^Gender: M = male, F = female; AIS = ASIA Impairment Scale: A = no motor or sensory function is preserved below the neurological level, B = sensory function is preserved but motor function is not preserved below the neurological level, C = Motor function is preserved below the neurological level and more than half of key muscle functions below the single neurological level of injury have a muscle grade less than 3/5 and D = Motor Incomplete. Motor function is preserved below the neurological level and at least half of key muscle functions below the neurological levle of injury have a muscle grade > or = 3/5; ASIA = American Spinal Injury Association; WUSPI = Wheelchair User's Shoulder Pain Index.

**Table 2:** Description of self-selected comfortable propulsion speed, experimental tasks completed, and rate of perceived exertion.

Participants	Self-selected speed (m/s)	Slopes					Rate of perceived exertion (/10)				
		0°	2.7°	3.6°	4.8°	7.1°	0°	2.7°	3.6°	4.8°	7.1°	
1	1.11	✓	✓	✓	✓	✓	0.9	7.4	5.5	8.1	9.5	
2	1.65	✓	✓	✓	✓		0.1	2.2	0.3	0.3	—	
3	1.05	✓	✓	✓	✓	✓	1.3	1.3	3.9	4.4	7.1	
4	1.18	✓	✓	✓	✓	✓	0.8	1.7	3.7	4.8	7.8	
5	1.20	✓	✓	✓	✓	✓	1.7	4.8	2.5	5.9	7.9	
6	0.91	✓	✓	✓			3.0	6.3	7.7	—	—	
7	1.48	✓	✓				0.9	9.7	—	—	—	
8	1.16	✓	✓	✓			0	0.3	0.9	—	—	
9	1.04	✓	✓	✓	✓		1.8	3.8	3.3	4.2	—	
10	1.27	✓	✓	✓	✓	✓	0	0	0	0	0	
11	1.39	✓	✓	✓	✓	✓	1.0	1.5	3.3	2.7	3.2	
12	0.99	✓	✓	✓	✓		1.1	4.1	4.0	7.5	—	
13	1.06	✓	✓				4.2	8.8	—	—	—	
14	1.25	✓	✓	✓	✓	✓	1.2	5.2	6.7	6.4	8.5	
15	1.03	✓	✓	✓	✓		0.0	0.6	1.9	3.6	—	
16	1.07	✓	✓	✓	✓	✓	0.7	3.4	4.6	6.6	9.2	
17	1.06	✓	✓	✓	✓	✓	0.7	6.2	2.6	6.5	8.9	
18	1.11	✓	✓	✓	✓	✓	2.9	4.6	6.1	6.6	7.3	

Mean	** 1.17**	*n* = **18**	*n* = **18**	*n* = **16**	*n* = **14**	*n* = **10**	**1.2**	**3.9**	**3.5**	**4.7**	**6.8**	
SD	**0.18**						**1.1**	**2.9**	**2.2**	**2.5**	**3.0**	

**Table 3:** Group average (SD) mean temporal parameter measured of the push phase measured at the nondominant handrim at the five different slopes tested.

	Slopes				
	0°	2.7°	3.6°	4.8°	7.1°
	Temporal parameters (s)				
Push phase	0.48 ± 0.08	0.48 ± 0.08	0.49 ± 0.08	0.48 ± 0.07	0.48 ± 0.06
Recovery phase	0.59 ± 0.22	0.27 ± 0.10	0.26 ± 0.09	0.22 ± 0.08	0.18 ± 0.05

Total (cycle)	1.07 ± 0.23	0.75 ± 0.16	0.75 ± 0.14	0.70 ± 0.13	0.66 ± 0.10

**Table 4:** Group average (SD), total excursion (°), and maximum and minimum (°) kinematic values measured at the trunk and nondominant shoulder at the five different slopes tested as well as the results of the ANOVA and post hoc comparison test.

		Slopes					ANOVA	Eta-squared	Post hoc comparisons			
		0°	2.7°	3.6°	4.8°	71°			0° versus 2.7°	2.7° versus 3.6°	3.6° versus 4.8°	4.8° versus 7.1°
Joint angle (°)		Trunk—flexion (+), extension (−)							Trunk—flexion (+), extension (−)			
	Excursion	5.88 (2.12)	12.67 (3.40)	14.56 (2.77)	17.61 (3.96)	22.37 (6.00)	<0.001	0.907	<0.001	0.006	<0.001	0.003
	Maximum	19.27 (6.23)	35.92 (8.45)	39.45 (8.31)	45.79 (8.53)	60.93 (11.30)	<0.001	0.889	<0.001	0.031	0.002	0.005
	Minimum	13.39 (6.14)	23.26 (7.75)	24.90 (8.05)	28.18 (7.71)	38.57 (8.59)	<0.001	0.910	<0.001	0.028	<0.001	<0.001
		Shoulder—flexion (+), extension (−)							Shoulder—flexion (+), extension (−)			
	Excursion	55.67 (9.20)	58.17 (10.92)	59.63 (9.01)	60.19 (6.98)	64.99 (8.95)	0.019	0.296	0.072	0.183	0.473	0.217
	Maximum	11.31 (9.34)	18.00 (8.20)	19.35 (7.62)	22.59 (7.30)	30.88 (7.45)	<0.001	0.849	<0.001	0.001	0.054	<0.001
	Minimum	−44.36 (9.35)	−40.17 (9.36)	−40.27 (9.15)	−37.60 (8.34)	−34.10 (10.36)	<0.001	0.651	<0.001	0.006	<0.001	<0.001
		Shoulder—abduction (+), adduction (−)							Shoulder—abduction (+), adduction (−)			
	Excursion	9.35 (3.43)	12.27 (7.16)	13.48 (6.61)	14.88 (7.56)	19.65 (11.88)	0.012	0.337	0.010	0.363	0.158	0.047
	Maximum	33.25 (8.00)	34.81 (10.65)	35.40 (10.26)	34.88 (10.57)	40.60 (15.43)	0.083	—	—	—	—	—
	Minimum	23.90 (6.27)	22.53 (7.26)	21.92 (6.66)	20.00 (5.45)	20.94 (5.70)	0.038	—	—	—	—	—
		Shoulder—internal rotation (+), external rotation (−)							Shoulder—internal rotation (+), external rotation (−)			
	Excursion	24.86 (8.20)	30.55 (10.24)	33.35 (8.83)	34.52 (9.44)	43.28 (11.34)	<0.001	0.623	0.004	0.040	0.765	0.013
	Maximum	31.57 (16.82)	37.89 (18.46)	40.15 (19.17)	43.02 (12.23)	49.69 (8.71)	<0.001	0.665	<0.001	0.029	0.855	0.033
	Minimum	6.71 (13.57)	7.34 (15.10)	6.80 (16.94)	8.50 (9.18)	6.41 (7.70)	0.152	—	—	—	—	—

**Table 5:** Group average (SD) mean and peak weight-normalised moments (Nm/kg) measured at the nondominant shoulder at the five different slopes as well as results of the ANOVA and post hoc comparison tests.

		Slopes					ANOVA	Eta-squared	Post hoc comparisons			
		0°	2.7°	3.6°	4.8°	71°			0° versus 2.7°	2.7° versus 3.6°	3.6° versus 4.8°	4.8° versus 7.1°
Normalised moments (Nm/kg)		Shoulder—flexion (+), extension (−)							Shoulder—flexion (+), extension (−)			
	Mean	0.140 (0.056)	0.226 (0.067)	0.248 (0.081)	0.233 (0.083)	0.242 (0.151)	0.027	0.288	<0.001	0.003	0.433	0.966
	Peak (maximum)	0.292 (0.109)	0.512 (0.152)	0.546 (0.167)	0.559 (0.128)	0.733 (0.302)	<0.001	0.588	<0.001	0.004	0.867	0.049
		Shoulder—abduction (+), adduction (−)							Shoulder—adduction (+), abduction (−)			
	Mean	−0.016 (0.023)	−0.060 (0.050)	−0.069 (0.046)	−0.090 (0.043)	−0.103 (0.036)	<0.001	0.623	<0.001	0.414	0.147	0.366
	Peak (minimum)	−0.088 (0.041)	−0.257 (0.105)	−0.278 (0.104)	−0.369 (0.137)	−0.494 (0.133)	<0.001	0.797	<0.001	0.149	0.002	0.002
		Shoulder—internal rotation (+), external rotation (−)							Shoulder—internal rotation (+), external rotation (−)			
	Mean	0.097 (0.036)	0.189 (0.050)	0.222 (0.064)	0.252 (0.065)	0.344 (0.101)	<0.001	0.726	<0.001	0.004	0.191	0.010
	Peak (maximum)	0.196 (0.078)	0.415 (0.144)	0.456 (0.129)	0.530 (0.148)	0.780 (0.184)	<0.001	0.801	<0.001	0.001	0.103	0.001

**Table 6:** Group average (1 SD), mean muscle utilisation ratio (MUR), peak MUR (/1), and indicator of muscle work (IMW) values for each muscle assessed at the nondominant shoulder at five different slope angles as well as results of the ANOVA and post hoc comparison tests.

	Slopes					ANOVA	Eta-squared	Post hoc comparisons			
	0°	2.7°	3.6°	4.8°	71°			0° versus 2.7°	2.7° versus 3.6°	3.6° versus 4.8°	4.8° versus 7.1°
	Anterior deltoid							Anterior deltoid			
Mean MUR (/1)	0.07 (0.04)	0.21 (0.09)	0.23 (0.12)	0.26 (0.10)	0.34 (0.13)	<0.001	0.831	<0.001	0.002	<0.001	0.001
Peak MUR (/1)	0.14 (0.08)	0.44 (0.23)	0.48 (0.26)	0.54 (0.26)	0.68 (0.28)	<0.001	0.836	<0.001	0.002	<0.001	<0.001
IMW	7.18 (3.98)	20.70 (9.43)	23.48 (12.37)	26.06 (10.43)	34.48 (13.04)	<0.001	0.829	<0.001	0.003	<0.001	0.002

	Posterior deltoid							Posterior deltoid			
Mean MUR (/1)	0.08 (0.07)	0.20 (0.14)	0.24 (0.24)	0.23 (0.10)	0.27 (0.06)	<0.001	0.907	<0.001	0.144	<0.001	<0.001
Peak MUR (/1)	0.20 (0.14)	0.52 (0.31)	0.62 (0.51)	0.60 (0.19)	0.77 (0.19)	<0.001	0.894	<0.001	0.027	0.001	0.005
IMW	7.95 (6.92)	20.36 (13.84)	24.43 (23.81)	23.35 (10.47)	27.34 (6.48)	<0.001	0.906	<0.001	0.143	<0.001	<0.001

	Clavicular fibers of pectoralis major							Clavicular fibers of pectoralis major			
Mean MUR (/1)	0.07 (0.04)	0.18 (0.10)	0.20 (0.10)	0.27 (0.14)	0.35 (0.18)	<0.001	0.754	<0.001	0.003	<0.001	0.005
Peak MUR (/1)	0.14 (0.09)	0.38 (0.22)	0.42 (0.23)	0.57 (0.31)	0.71 (0.29)	<0.001	0.814	<0.001	0.009	<0.001	0.001
IMW	7.00 (4.26)	18.37 (9.77)	20.41 (10.46)	27.04 (14.32)	35.28 (17.57)	<0.001	0.752	<0.001	0.003	<0.001	0.005

	Sternal fibers of pectoralis major							Sternal fibers of pectoralis major			
Mean MUR (/1)	0.12 (0.08)	0.30 (0.15)	0.32 (0.13)	0.37 (0.15)	0.53 (0.24)	<0.001	0.781	<0.001	0.005	0.001	0.006
Peak MUR (/1)	0.21 (0.15)	0.54 (0.23)	0.59 (0.23)	0.71 (0.26)	1.01 (0.42)	<0.001	0.829	<0.001	0.003	<0.001	0.002
IMW	11.94 (7.53)	30.23 (14.77)	32.27 (13.23)	37.53 (15.11)	52.80 (24.06)	<0.001	0.780	<0.001	0.005	0.001	0.006

## Abbreviations

AIS: American Spinal Injury Association Impairment Scale
EMG: Electromyography
ER: External rotation
LED: Light-emitting diode
IMW: Indicator of muscle work
IR: Internal rotation
JCS: Joint coordinate system
MUR: Muscle utilization ratio
MVC: Maximum voluntary contraction
MWC: Manual wheelchair
U/L: Upper limb
WUSPI: Wheelchair User's Shoulder Pain Index.
